# Hematological Malignancy in a Hypophysectomised Acromegalic Patient Under 4-Year Therapy with Somatostatin Analogues: From a Rib Lump Underlying Bone Plasmatocytoma Features to Multiple Myeloma

**DOI:** 10.3390/diagnostics15202623

**Published:** 2025-10-17

**Authors:** Mihaela Stanciu, Alina Cătană, Ruxandra Paula Ristea, Denisa Tanasescu, Mara Carsote, Florina Ligia Popa, Ioana-Codruța Lebădă

**Affiliations:** 1 Department of Endocrinology, Faculty of Medicine, “Lucian Blaga” University of Sibiu, 550024 Sibiu, Romania; mihaela.stanciu@ulbsibiu.ro (M.S.); codruta.lebada@ulbsibiu.ro (I.-C.L.); 2 Department of Endocrinology, Clinical County Emergency Hospital, 550245 Sibiu, Romania; ruxandraszebin@yahoo.com; 3 Department of Haematology, County Clinical Emergency Hospital of Sibiu, 550245 Sibiu, Romania; alinabrabete@yahoo.com; 4 Medical Clinical Department, Faculty of Medicine, “Lucian Blaga” University of Sibiu, 550024 Sibiu, Romania; 5 Department of Endocrinology, “Carol Davila” University of Medicine and Pharmacy, 020021 Bucharest, Romania; 6 Department of Clinical Endocrinology V, “C.I. Parhon” National Institute of Endocrinology, 011863 Bucharest, Romania; 7 Department of Physical Medicine and Rehabilitation, Faculty of Medicine, “Lucian Blaga” University of Sibiu, 550024 Sibiu, Romania; florina-ligia.popa@ulbsibiu.ro

**Keywords:** acromegaly, myeloma, endocrine, hormone, IGF1, octreotide, lanreotide, MRI, core biopsy, immunohistochemistry

## Abstract

Acromegaly is associated with a higher risk of certain malignancies, but not hematological neoplasia, although multiple myeloma (MM) was found in very limited cases. We aim to present such a case, adding a particular presentation with co-occurrence of a plasmocytoma. A 52-year-old male with acromegaly confirmed at 46 (MRI: pituitary macroadenoma of 12 × 11 × 10 mm) underwent hypophysectomy followed by 3 years of octreotide LAR then lanreotide depot. After another 6 months, he experienced a rapidly growing, painful lump in the right lateral thoracic area confirmed by CT as a 9-cm osteolytic lesion at the third rib. Core biopsy revealed plasmocytoma of the bone and medullary biopsy confirmed MM. Plasmacytoma was managed with 10 radiotherapy sessions, with favorable outcome and mass resorption; MM was managed with a VRD regimen, followed by autologous hematopoietic stem-cell transplantation. Six months after sFLC normalization and plasmacytoma resorption, complete remission was reported. In the meantime, lanreotide was continued, with complete acromegaly control. To conclude, what started as a rather typical scenario for an otherwise rare condition, as is acromegaly in the general population (but not so rare for endocrinologists), turned into an unexpected and more severe outcome. Noting this exceptional association, we pinpoint that further research is needed for understanding the dual acromegaly–MM relationship.

**Figure 1:**
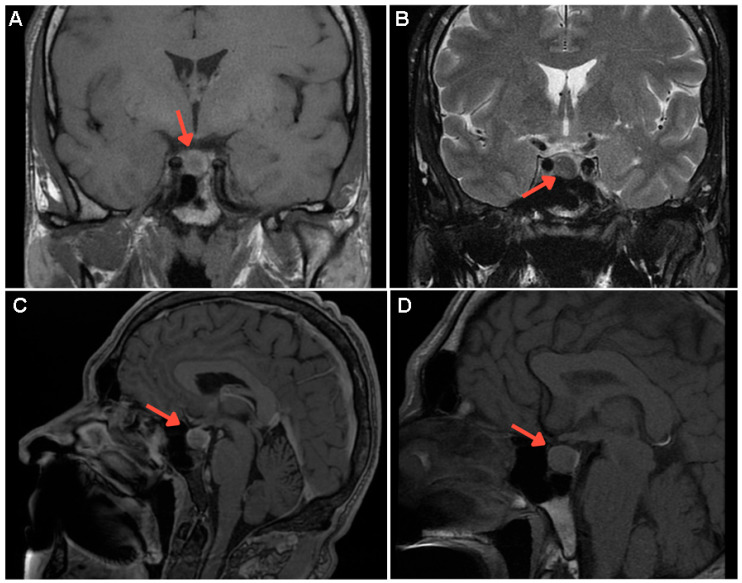
This case study involved a 52-year-old non-smoking Caucasian male with acromegaly confirmed at the age of 46. The initial diagnosis had been established based on a highly suggestive clinical presentation (acromegalic facies with hypertrophy of the frontal bone, enlarged nose, thickened lips and ears, prognathism, increased incisor spacing, and macroglossia, as well as hand and foot enlargement, hyperhidrosis, persistent headache, joint pain, dorsal kyphosis, and arterial hypertension). Many of these elements had been self-observed within the previous two years with a progressive appearance. On admission, insulin-like growth factor (IGF-1—chemiluminescent assay) was four times above the normal upper limit, with unsuppressed growth hormone (GH) identified during 75-g oral glucose tolerance test (OGTT) and a GH nadir of 18.7 (normal <0.4—ultrasensitive chemiluminescent assay) ng/mL (Table S1). His medical family history was negative. Contrast-enhanced magnetic resonance imagining (MRI) showed a pituitary macroadenoma of 12 by 11 by 10 mm, without optic chiasma involvement. [(**A**) Coronal T1-weighted image: well-defined, inhomogeneous tumour mass (red arrow) in the para-median right pituitary area; (**B**) Coronal T2-weighted pituitary image: isointense tumour features (red arrow); (**C**,**D**) Post-contrast sagittal T1-weighted image: non-enhanced tumour mass at different section levels (red arrow)].

**Figure 2:**
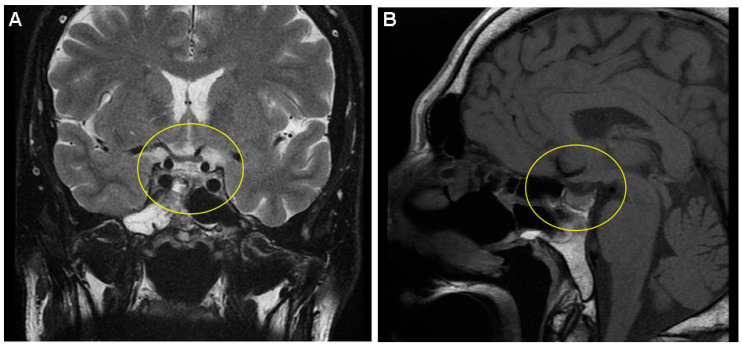
As a first-line approach, the patient underwent an endoscopic endo-nasal trans-sphenoid resection of the pituitary tumour with sellar dura reconstruction using a homologous fascia lata graft. No intra- or post-operatory complications were registered, except for mild hypopituitarism that required replacement for a few months (daily oral prednisone 5 mg and levothyroxine 25 µg). Pathological analysis confirmed a pituitary adenoma (no atypia at hematoxylin–eosin stain). In addition, immunohistochemistry revealed negative chromogranin A and a Ki67 index of proliferation of <1%, while CKAE1/AE3 was granularly positive intra-cytoplasmic and para-nuclear (no other pituitary hormone was positive). Post-hypophysectomy, no tumour remnant was found via MRI ((**A**) Coronal T1-weighted image; (**B**) Sagittal T1-weighted image). The hormonal assays confirmed active disease (with discrepancies between a normal IGF-1 value and an abnormal OGTT-GH nadir) (Table S1). Octreotide long-acting release (LAR) (s.c. 20 mg, every 28 days) was offered for 36 months, which controlled the hormonal excess, while annual MRI showed stationary aspects. Then, the somatostatin analogue was stopped for 2 months (according to the country’s protocol of free reimbursement) followed by drug re-initiation due to an OGTT-GH nadir of 1.25 (normal: <0.4) ng/mL associated with normal IGF-1, this time with lanreotide depot (s.c. 120 mg, every 42 days). Also, a check-up colonoscopy was performed and found to be normal.

**Figure 3:**
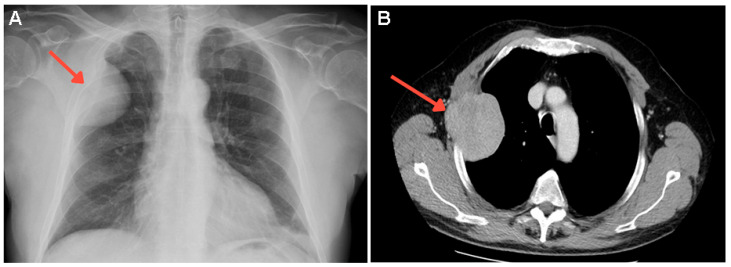
Almost 4 years after initial acromegaly diagnosis, the subject experienced a rapidly growing, painful lump in the right lateral thoracic area. X-ray identified a large, nodular opacity of 7.3 cm, located in the right upper latero-thoracic chest wall (red arrow) (**A**). Thoracic contrast-enhanced computed tomography (CECT) revealed an osteolytic lesion of 9 cm (red arrow) invading the third right rib (**B**). At that point, he was undergoing lanreotide 120 mg every 42 days for 6 months. The endocrine assessment showed normal IGF-1, but the nadir of OGTT-GH was 1.29 ng/mL, suggesting suboptimal disease control. Otherwise, the biochemical and hormonal panel was normal, including total serum calcium, total alkaline phosphatase, renal function, and parathyroid hormone (PTH) (Table S1).

**Figure 4:**
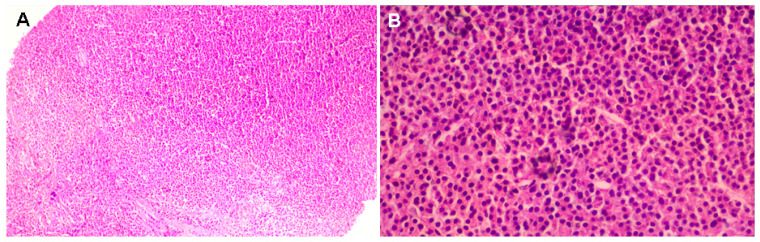
A core biopsy was performed and histological analysis showed tissue fragments displaying solid tumour proliferation, characterized by focal nests with incomplete septae, pseudo-rosettes, and trabecular structures: small, round cells with eosinophilic cytoplasm and round nuclei. The mitotic activity was moderate, with 5 mitoses per 10 high-power fields, without evidence of necrosis. [(**A**) Hematoxylin–eosin stain: hyper-cellularity with plasma cells (magnification 10×); (**B**) Hematoxylin–eosin stain: round plasma cells with round nuclei, some with visible nucleoli and dispersed chromatin (magnification 40×)].

**Figure 5:**
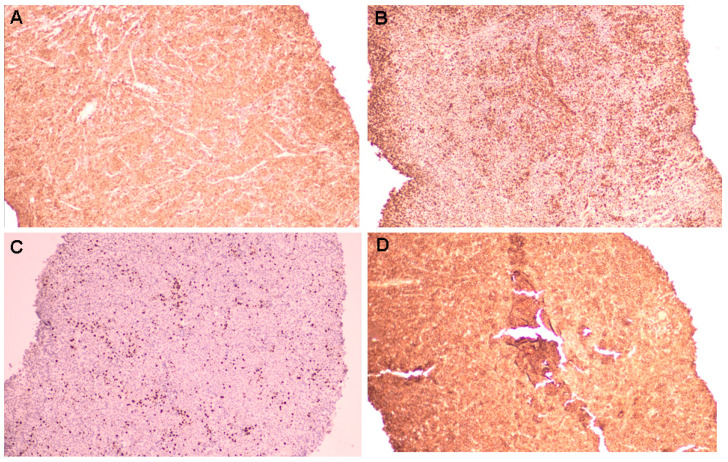
Immunohistochemistry analysis revealed positive BCL2 ((**A**) magnification 10×) and vimentin ((**B**) magnification 10×) expression. Ki67 was 35% ((**C**) magnification 10×). The tumour showed strong expression of CD138 ((**D**) magnification 10×), which is a characteristic marker of plasmacytoma. Additional immunostaining was performed for differential diagnosis (and found negative), specifically CD168, S100, HMB45, chromogranin, and synaptophysin. All these sustained a diagnosis of plasmocytoma of the bone.

**Figure 6:**
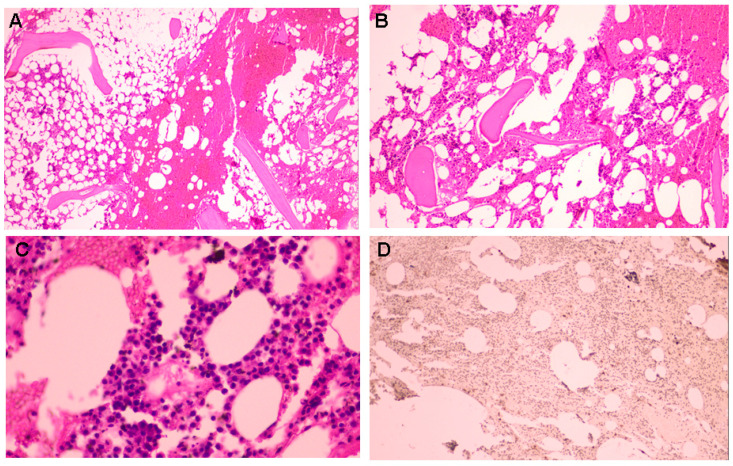
A medullary biopsy was performed from the iliac bone, revealing cellularity ranging from 35% to 40%, with irregular distribution. The histological report showed an interstitial infiltration characterized by large plasma cells with prominent cytoplasms. These cells exhibited nuclei resembling those observed in plasmacytomas, with some of them containing nucleoli. Immunohistochemistry analysis highlighted a CD138-positive reaction. [(**A**) Hematoxylin–eosin stain: hyper-cellular marrow with significant cellularity (magnification 4×); (**B**) Hematoxylin–eosin stain: higher magnification revealed an irregularly distributed hyper-cellular marrow (magnification 10×); (**C**) Hematoxylin–eosin stain: large plasma cells with prominent cytoplasms in an interstitial pattern (magnification 40×); (**D**) Immunohistochemistry: positive assay for CD138 (magnification 10×)]. Serum-free light chain (sFLC) lambda was increased at 1686 mg/dL, with a kappa of 10.4 mg/L and a kappa/L ratio of 0.01 (Table S2). Bone marrow aspiration revealed rare plasma cells with hemosiderin granules in the cytoplasm, and sideroblasts accounting for 43% of the observed cells. May–Grunwald Giemsa (MGG)-stained myelogram showed high cellularity, with an uneven distribution of the myeloid series. Areas of infiltration by plasma cells were clearly detectable, characterized by cellular anisocytosis, high basophilic cytoplasm, and central or eccentric nuclei with lax chromatin, with a variable distribution ranging from 36% to 72%. Bence Jones proteinuria was negative. Thus, the patient was confirmed with multiple myeloma—stage III A Lambda Multiple Myeloma according to Durie–Salmon staging and stage II according to the International Staging System (ISS). CRAB (hypercalcemia, renal failure, anemia, or lytic bone lesions) features, hepato-splenomegaly, lymphadenopathies, and infections were ruled out. No other abnormalities were detected via positron emission tomography/computed tomography (PET/CT) or whole-body diffusion-weighted magnetic resonance imaging (DW-MRI).

**Figure 7:**
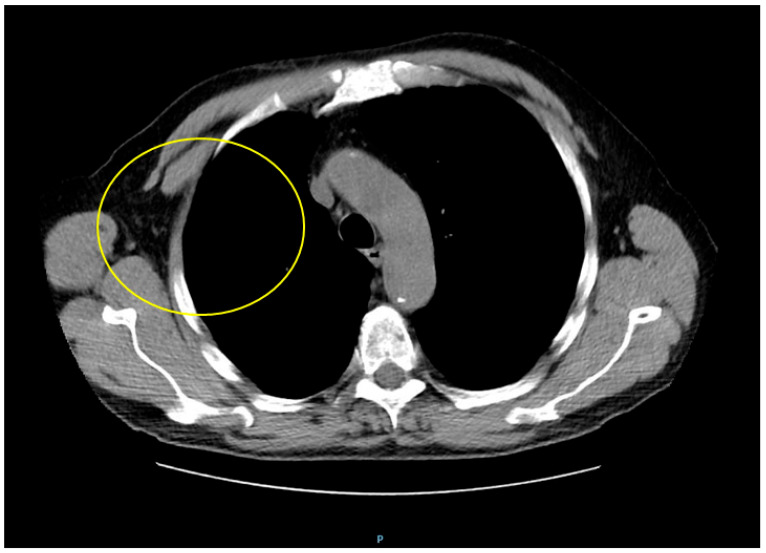
The plasmacytoma of the bone was managed with 10 radiotherapy sessions, resulting in a favorable outcome with mass resorption (capture shows thoracic computed tomography scan: complete resorption of the plasmacytoma after radiotherapy with osteolysis of the third right rib). Multiple myeloma was managed with lenalidomide, low-dose dexamethasone, and bortezomib (VRD regimen). Lenalidomide was administered after radiotherapy, but was interrupted after the patient presented a skin rash following the first two sessions. Then, he underwent autologous hematopoietic stem-cell transplantation (ASCT). Adjunctive treatment included bisphosphonate at chemotherapy initiation (zoledronic acid) and analgesics and thrombophilaxy during lenalidomide treatment. At 6 months of follow-up after the normalization of sFLC and immunoglobulin (Ig) M, alongside plasmacytoma resorption, complete remission was reported (Table S2). In the meantime, the patient continued lanreotide depot 120 mg every 28 days, which achieved complete acromegaly control (Table S1 and Figure S1). Overall, 95% to 98% of all acromegaly cases are caused by a GH-producing pituitary tumour. The prevalence in the general population is low (40 to 70 cases in a million), with an annual incidence of 3–4 cases in a million [1,2,3,4]. At the moment of clinical recognition, a 10-year history of unrecognized disease (but associated with persistently elevated IGF-1/GH levels, potentially causing multi-organ complications) is involved [5,6,7], as probably found in this case. **Cancer-related death** is the third leading cause (after cardiovascular diseases and respiratory insufficiency) in acromegalic individuals, and is generally associated with a 1.6- to 3.3-fold increase in age-related mortality as well as a 10-year decrease in life expectancy, according to some analyses (overall, almost 15% to 25% of these patients die of oncologic conditions). The most common malignancy is colonic cancer, followed by (reported with various rates and not unanimously agreed) breast, thyroid, prostate carcinomas and melanoma. IGF-1 (and, to a lesser, extent GH) is involved in the cells proliferation, differentiation, metabolism, apoptosis, as well as angiogenesis [7,8,9,10]. However, hematological malignancies are exceptional in acromegaly [11], and only a few cases of multiple myeloma have been published so far [12,13,14,15,16,17,18,19]. **Multiple myeloma**, a malignant proliferative disease of the B-cells, accounts for approximately 1% of all cancers and about 10% of all hematological malignancies, with an age-related increasing incidence and a median at diagnosis of 68 years. The disease is characterized by an overproduction of monoclonal plasma cells in the bone marrow (clonal plasmocytosis should be at least 15% on bone marrow examination) that also leads to elevated monoclonal proteins in the blood and urine [20,21,22]. Quite the opposite from a prevalence perspective, **plasmacytoma**, either solitary or extramedullary, is a very rare plasma cell anomaly that targets the axial skeleton and soft tissues without causing systemic symptoms, being regarded as a precursor to plasma cell malignancies. The risk of progression to multiple myeloma is 65% to 85% within 10 years [23,24]. Plasmocytoma was reported as a single bone tumour more frequent in men, typically at 55 years or older. The first clinical element is usual local (skeletal) pain, as reported in this patient [23,24,25]. **Potential pathogenic connections** between acromegaly and multiple myeloma (which are still an open matter, and none of which is entirely conclusive so far) include: 1. GH and IGF-1 might play a role in B-lymphocyte activation; 2. a universal expression of the IGF-1 receptor (IGF-1R) was found in multiple myeloma cells in vitro. IGF-1 has stimulatory effects in some of these cell lines, probably in the later stages of B-cell development; 3. acromegaly is considered to induce the progression of monoclonal gammapathy of undetermined significance (MGUS) into multiple myeloma, according to two previous case reports [17,18]; 4. IGF-1 administration to plasma tumour cell lines doubles their growth rate (animal studies); 5. generally, as mentioned, IGF-1 and GH promote proliferation and inhibit the apoptosis of cancer cells (this being a clearly recognized pathogenic mechanism in acromegaly-related oncologic ailments). Moreover, GH might dysregulate the IGF-1-to-IGFBP-3 (insulin-like growth factor binding protein type 3) ratio, noting that IGF-1 acts as a mitogen while IGFBP-3 promotes cell apoptosis; 6. additionally, experimental models have showed that GH administration might stimulate B-cell proliferation, suggesting direct intervention of GH (of note, in our case, IGF-1 remained normal, but GH was mildly increased more than 3 years after surgery); 7. hormonal excess in acromegaly might cause paracrine and autocrine anomalies as well, specifically in the immune system; 8. IGF-1 may contribute to B-cell development, which further promotes myeloma development via interleukin-6 cascade (murine experiments); 9. it is unclear if GH-induced insulin resistance is a contributor to B-cell anomalies (in the present case, the patient was not diabetic); 10. insulin receptor substrate 1 (downstream of IGF-1R) activates phosphatidylinositol-3′-kinase (PI-3K), which activates either the Akt–Bad signal transduction pathway (prone to apoptosis inhibition) or mitogen-activated protein kinase (MAPK), promoting cell proliferation, as observed in multiple myeloma cell lines [14,15,16,26,27]. We found no specific studies/reports to address the co-diagnosis of acromegaly and **plasmocytoma**, except for one report (from 2012) that detected a pituitary tumour mass upon 18FDG (fluorodeoxyglucose)-PET/CT screening after an initial diagnosis of plasmocytoma. Then, the same imaging method was used to supervise the endocrine tumour under lanreotide therapy [28,29]. In our case, the plasmocytoma of the bone should be assimilated into the complex landscape of multiple myeloma, but still received a specific therapeutic approach in terms of radiotherapy. Whether IGF-1/GH represented a risk or accelerating factor for myeloma is still of indeterminate significance. We also mention that multiple myeloma might manifest with **hypercalcemia**, which otherwise is typically found in acromegaly in association with primary hyperparathyroidism [multiple endocrine neoplasia (MEN) type 1 and type 4 in individuals harboring pathogenic variants of MEN1/menin and CDKN1B (cyclin-dependent kinase inhibitor) genes, respectively], or with humoral hypercalcemia of malignancy, as identified, for instance, in simultaneous bone metastatic cancers with suppressed PTH or in non-metastatic disease with PTH-related peptide (PTHrP) over-secretion [30,31]. In this case, the mineral metabolism assays were normal (of note, this is a real-life setting, and the entire panel of bone turnover markers was not available, except for total alkaline phosphatase). In addition, we mention a prior published case from 2019 presenting not only acromegaly and multiple myeloma, but also gastrointestinal stromal tumour (GIST), non-small cell lung carcinoma, clear cell renal carcinoma and benign endocrine tumors at the level of the adrenal cortex and thyroid. The typical gene panel for endocrine tumors/cancers was negative, as well as the screening based on a next-generation cancer panel for 94 cancer genes [19]. The **genetic** constellation in this subgroup of patients remains unknown. Noting that the first report of acromegaly and multiple myeloma in Asia was published in 2015 (in a 58-year-old female) [14], we mention a nationwide population-based cohort study in Korea that was published in 2025 (2382 acromegalic patients and 11,910 controls), which is the single study we could identify that showed a statistically significant higher risk for multiple myeloma in acromegalic patients (hazard ratio of 3.38, 95% confidence interval between 1.22 and 9.35) [32]. This raises the issue of certain population subgroups that could be more exposed to B-cell anomalies amid GH-producing tumors, which is yet to be explored. Notably, acromegaly includes a larger spectrum of other **bone deformities** (e.g., kyphosis and acral enlargement, as described in this case). On the other hand, amyloidosis with multiple myeloma (both conditions being in the same landscape of clonal plasma cell anomalies) at the level of the facial bones might mimic acromegalic features [33]. **The timeline** of diagnosis in prior reports pinpointed either a synchronous diagnosis of acromegaly and multiple myeloma (at the moment where the hormonal excess was clearly confirmed) [13,14], or with a gap of 2 [19] or 5 years [16]. However, one of the earliest reports showed a 9-year gap between acromegaly recognition and an IgG kappa myeloma diagnosis in a female in her 60s [12]. As mentioned, suboptimal control of GH and IGF-1 normalization, associated with clinically stable disease, was found at rib lump identification in the present case. Generally, 20% of acromegaly cases show biochemical discrepancies in IGF-1 versus GH testing and this might become a pitfall in achieving remission under medical therapy [34]. Interestingly, the patient underwent 4 years of **somatostatin analogues**, and to our best knowledge, there are limited data to report this specific type of management before myeloma confirmation [16,19], and neither of the previous cases also showed a plasmocytoma (in addition, none of these cases reported the application of ASCT). This therapy is well recognized as an active anti-proliferative tool to address neuroendocrine neoplasia through different configurations of the five somatostatin receptors, which might also interfere with the immune system, and earlier studies suggested interference with human multiple myeloma cells via interleukin-6 [35,36]. This is part of a larger neuroendocrine frame in the immunolymphoproliferative diseases, a subchapter to be explored with respect to the association above. The primary factor to decide the outcome remains multiple myeloma, but the acromegaly-related disease burden worsens the prognosis. However, the prognostic factors and long-term outcomes are uncertain due to the lack of standardized therapeutic protocols and specific indicators of risk assessment. **To conclude**, what started as a rather typical scenario for an otherwise rare condition, as is acromegaly in the general population (but not so rare for endocrinologists) [1,2,3,4], turned into an unexpected and more severe outcome. Noting this exceptional association, we pinpoint that further research is needed for the understanding of the dual relationship between acromegaly and multiple myeloma (common pathogenic loops or incidental overlapping?). Practical bases of multimodal management are yet to be clarified, since there are no specific guidelines in this instance to indicate a particular therapy regime or new targets of GH/IGF-1 levels to be chosen. Whether there is a distinct subgroup of acromegalic patients who are prone to hematologic malignancies in terms of genetic, epigenetic, and hormonal (including population-based) contributors is still to be determined. Plasmocytoma might represent an “intermediary” step to multiple myeloma, and GH/IGF-1 excess should be regarded as a trigger or an accelerating factor. The epidemiologic impact of this unusual association remains at a few documented case reports (Table S3) and awareness represents the key operating factor.

## Data Availability

The original contributions presented in this study are included in the article/Supplementary Materials. Further inquiries can be directed to the corresponding author.

## Supplementary Materials

The following supporting information can be downloaded at: https://www.mdpi.com/article/10.3390/diagnostics15202623/s1, Figure S1: Timeline perspective of the case; Table S1: Hormonal assays after acromegaly diagnosis; Table S2: Hematologic profile at multiple myeloma diagnosis and after autologous hematopoietic stem-cell transplantation. Table S3: Review of literature; The method of search included PubMed and Clarivate/WOS databases with no time line restriction, neither study design limitations of freely available articles and/or abstract, upon using various combinations of key search words (including “acromegaly”, “growth hormone”, “hematological”, “myeloma”, “plasmocytoma”, etc.); the display starts with the most recent publication date. All the data provided by this analysis are in the main text.

## Abbreviations

The following abbreviations are used in this manuscript:

ACTH	adrenocorticotropic hormone
ASCT	autologous hematopoietic stem-cell transplantation
cm	centimetre
CECT	contrast-enhanced computed tomography
CRAB	hypercalcaemia, renal failure, anaemia, lytic bone lesions
CDKN1B	cyclin-dependent kinase inhibitor
DW-MRI	whole-body diffusion-weighted magnetic resonance imaging.
FSH	follicle-stimulating hormone
FDG	fluorodeoxyglucose
GH	growth hormone
GIST	gastrointestinal stromal tumour
IGF-1	insulin-like growth factor
IGF-1R	IGF-1 receptor
IGFBP-3	insulin-like growth factor binding protein type 3
Ig	immunoglobulin
LAR	long-acting release
LH	luteinizing hormone
MRI	magnetic resonance imagining
MGG	May–Grunwald Giemsa
MEN	Multiple Endocrine Neoplasia
MGUS	monoclonal gammapathy of undetermined significance
MAPK	mitogen activated protein kinase
NA	not available
OGTT	oral glucose tolerance test
PTH	parathyroid hormone
PTHrP	parathyroid hormone-related peptide
PET/CT	positron emission tomography/computed tomography
PI-3K	phosphatidylinositol-3′-kinase
s.c	subcutaneous
sFLC	serum-free light chain
TSH	thyroid stimulating hormone
T4	thyroxine
VRD	lenalidomide, low-dose dexamethasone, and bortezomib
