# Supplementary material for: Hematological Malignancy in a Hypophysectomised Acromegalic Patient Under 4-Year Therapy with Somatostatin Analogues: From a Rib Lump Underlying Bone Plasmatocytoma Features to Multiple Myeloma
Source: Diagnostics (Basel). 2025 Oct 17;15(20):2623. doi: 10.3390/diagnostics15202623 (PMC12563066; doi:10.3390/diagnostics15202623)
Supplement: Supplementary file 1 [file diagnostics-15-02623-s001.zip › diagnostics-3901162-supplementary.pdf]

Figure S1. Timeline perspective of the case

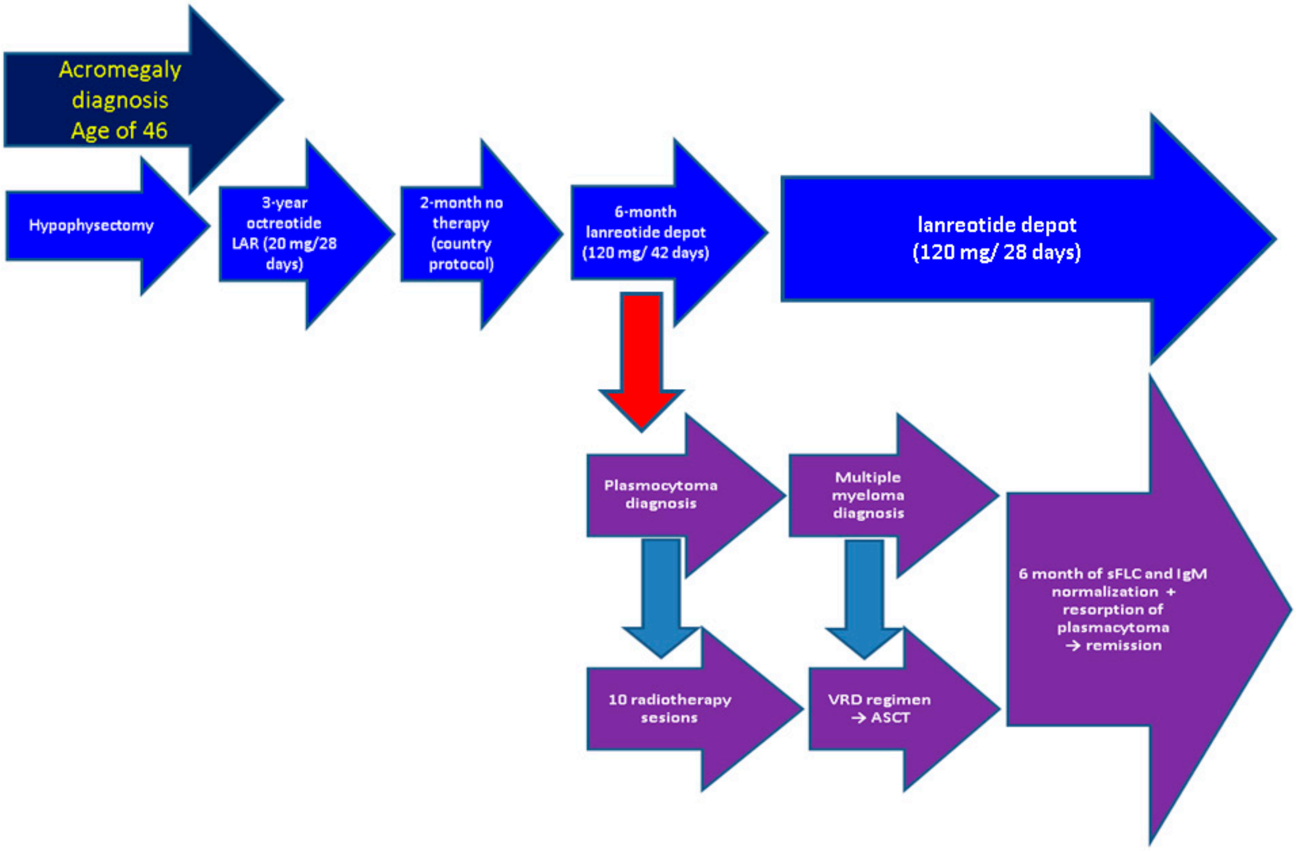

Table S1. Hormonal assays since acromegaly diagnosis

Biochemical or hormonal assay<br>(Unit)<br>(normal ranges)	At acromegaly diagnosis<br>(age of 46)	1 month after hypophysectomy	1 year under octreotide LAR 20 mg/28 days	3 years under octreotide LAR 20 mg/28 days	2 months without somatostatin analogue (according to the reimbursement protocol)→re-start with lanreotide	At lanreotide initiation	6 months under lanreotide depot (120 mg/42 days):<br>at rib lump identification*	1 year since transplant (under lanreotide depot 120 mg/28 days)*
GH-OGTT (ng/mL)<br>glycaemia (mg/dL) – baseline	8.93<br>107	15.2<br>91	0.12<br>105	0.191<br>96		1.43<br>80	0.954<br>83	3.48<br>101
GH-OGTT (ng/mL)<br>glycaemia (mg/dL) – 30′	18.7<br>168	5.06<br>138	0.08<br>131	0.396<br>168		1.66<br>171	1.43<br>173	0.52<br>146
GH-OGTT (ng/mL)<br>glycaemia (mg/dL) – 60′	35.4<br>112	3.2<br>96	0.12<br>191	0.295<br>168		1.32<br>145	1.66<br>146	0.47<br>112
GH-OGTT (ng/mL)<br>glycaemia (mg/dL) – 90′	29.4<br>107	1.7<br>107	0.25<br>180	0.220<br>91		NA	1.32<br>84	NA
GH-OGTT (ng/mL)<br>glycaemia (mg/dL) – 120′	23.2<br>87	1.4<br>109	0.16<br>59	0.238<br>39		1.25<br>60	1.29<br>57	0.69<br>37
IGF1 (ng/mL)<br>(80.6→209)	836.6	145	122	132.5		165	165.3	132.6
Prolactin (ng/mL)<br>(2.1→17.7)	12.95	9.57	11.73	11		11	12	10.2
FSH (mIU/mL)<br>(1.4→18.1)	33.95	33.17	34.8	33		34	NA	33
LH (mIU/mL)<br>(1.5→9.3)	5.58	3.36	9.01	8		9.01	NA	9

**total testosterone (ng/dL) (164→753)**	340	513	706.9	699	600	NA	500
**ACTH (pg/mL) (7.2 →63.3)**	11.23	10.41	15.2	13	14.76	NA	13
**baseline morning cortisol (μg/dL) (5.27→22.45)**	7.08	16.2	12.5	12.1	13.2	NA	12.3
**TSH (μUI/mL) (0.55→4.78)**	3	3.2	3	2.9	2.9	NA	2.8
**FreeT4 (ng/dL) (0.89→1.76)**	1.15	1.05	1.3	1.2	1.01	NA	1.08
**total serum calcium (mg/dL) (8.4→10.2)**	9.94	9.78	9.29	9.1	9.2	9.25	9.2
**PTH (pg/mL) (6→65)**	52	NA	NA	NA	NA	52	NA
**creatinine (mg/dL) (0.7→1.3)**	0.76	0.75	1.06	1.01	1.02	1.06	1.07
**urea (mg/dL) (19→44)**	24	34	32	29	31	34	35
**total alkaline phosphatase (IU/L) (40→150)**	88	NA	78	76	NA	47	50

Abbreviations: ACTH = adrenocorticotropic hormone; FSH = follicle stimulating hormone; GH = growth hormone; IGF1 = insulin-like growth factor; LH = luteinizing hormone; NA = not available; OGTT = 75-gram oral glucose tolerance test; PTH = parathyroid hormone; TSH = thyroid stimulating hormone; T4 = thyroxine; red font = abnormal values or conclusive for the diagnosis; blue font = the patient underwent medication with octreotide LAR 20 mg/28 days; green font = the patient underwent medication with lanreotide depot 120 mg/42 days; *after this evaluation, the dose was increased to lanreotide depot 120 mg/28 days

Table S2. Hematologic profile at multiple myeloma diagnosis and after autologous hematopoietic stem-cell transplantation

Parameter	At diagnosis of multiple myeloma	After autologous hematopoietic stem-cell transplantation	Normal ranges	Units
**Hemoglobin**	14.4	13.5	13-17	g/dL
**Platelet count**	200	103	150-400	$10^3/\mu L$
**White blood cells count**	7.69	3.05	4-10	$10^3/\mu L$
**β2 microglobulin**	3.14	NA	2.5-7.2	%
**Serum quantitative IgG**	753	1078	700-1600	mg/dL
**Serum protein electrophoresis Albumin/globulin**	1.7	1.52	1-2	
**Serum IgM monoclonal protein**	60	31	40-230	mg/dL
**Kappa free light chain**	10.4	12.6	3.3-19.4	mg/dL
**lambda free light chain**	1686	88.9	5.71-26.3	mg/dL
**Free light chain ratio**	0.01	0.14	0.26-1.65	

Table S3. Review of literature; The method of search included PubMed and Clarivate/WOS databases with no time line restriction, neither study design limitations of freely available articles and/or abstract, upon using various combinations of key search words (including “acromegaly”, “growth hormone”, ”hematological”, “myeloma”, “plasmocytoma”, etc.); the display starts with the most recent publication date. All the data provided by this analysis are in the main text.

Reference number in the main text<br><br>First author<br><br>Publication year	Age of the patient	Gender	Timeline of diagnosis: acromegaly and hematologic malignancy	Type of hematologic neoplasia	Extramedullary tumor	Treatment for both diseases (including drug therapy, hypophysectomy, and autologous hematopoietic stem cell transplantation)	Disease control status	GH/IGF-1 excess as potential trigger for myeloma	Other malignant tumors, cardiovascular disease, and respiratory failure
[19]<br><br>Jawiarczyk-Przybyłowska et al.<br><br>2019	68	F	multiple myeloma was diagnosed 2 years since initial diagnosis of acromegaly	multiple myeloma with secondary renal amyloidosis	no	octreotide LAR (30 mg/month)<br>(the patient refused hypophysectomy)<br><br>chemotherapy with bortezomib + dexamethasone → switched to melphalan and prednisone due to nephrotic syndrome	controlled disease (no other outcomes)	yes	GIST<br><br>non-small cell lung carcinoma<br><br>clear cell renal carcinoma<br><br>benign tumors at adrenal cortex and thyroid<br><br>type 2 diabetes<br><br>hypertension<br><br>kidney stones<br><br>hepatic cyst<br><br>gallbladder polyps
[14]<br><br>Kang et al.<br><br>2015	58	F	multiple myeloma and acromegaly diagnosis was synchronous	multiple myeloma	no	hypophysectomy<br><br>follow-up for multiple myeloma (no chemotherapy)	acromegaly was not controlled at the moment of myeloma diagnosis → no other outcome data	yes	mild anemia<br><br>(probably sleep apnea) – originally described as "snoring" and "daytime drowsiness" with normal thyroid function
[13]<br><br>Atmaca et al.<br><br>2013	80	F	multiple myeloma and acromegaly diagnosis was synchronous	multiple myeloma	no	patient refused hypophysectomy and chemotherapy (VAD regimen was proposed)	acromegaly was not controlled at the moment of myeloma diagnosis	yes	hypertension<br>hypercalcemia with suppressed PTH<br>the patient died within a month from diagnosis due to pneumonia-related respiratory failure
[28], [29]<br><br>Maiza et al. 2014<br><br>Maiza et al. 2012	60	M	plasmocytoma and acromegaly diagnosis was synchronous	clavicular plasmocytoma	single plasmocytoma of bone	lanreotide (the dose was NA)<br><br>local radiotherapy for plasmocytoma (40 Gy in 20 fractions) without complementary chemotherapy	controlled acromegaly and no remission of the plasmocytoma during 4-month follow-up	yes	no
[16]<br><br>Barbosa et al.	59	F	multiple myeloma was diagnosed 5 years since	multiple myeloma	yes (lytic lesion at skull,	hypophysectomy → octreotide 150 μg/day for 11 months → switch to	acromegaly was controlled at the moment	no	pancytopenia<br><br>renal insufficiency

2011			initial diagnosis of acromegaly		clavicle, ribs, humeru s, femur, pelvis, thoracic and lumbar spine)	octreotide LAR 20 mg/month which achieved disease control → continued for 3 years (several times discontinuation due to failure of octreotide supply)<br><br>vincristine-adriamycin-dexamethasone (VAD) regimen	of myeloma diagnosis under octreotide LAR<br><br>clinical response to VAD regimen (a bine marrow transplantati on was planned)		
[18]<br><br>Tucci et al.<br><br>2009	60s	F	MGUS and acromegaly diagnosis was synchronous	MGUS transformed to multiple myeloma after 5 years since initial diagnosis	NA	NA	acromegaly was not controlled at the moment of myeloma transformati on	yes	NA
[17]<br><br>Kanazawa et al.<br><br>2006	65	F	MGUS and acromegaly diagnosis was synchronous	MGUS	NA	hypophysectomy<br><br>(no specific therapy for MGUS)<br><br>post-operatory cabergoline therapy	controlled both acromegaly and MGUS after hypophysect omy	yes	diabetes mellitus (insulin dependent)<br><br>spinal canal stenosis<br><br>carpal tunnel syndrome
[12]<br><br>Hägg et al.<br><br>1988	51	F	9-year gap between acromegaly diagnosis and multiple myeloma diagnosis	multiple myeloma	no	she declined any therapy for acromegaly for 6 years, then underwent radiotherapy	acromegaly was not controlled at the moment of myeloma diagnosis	yes	NA

[Additional abbreviations: F = female; M = male; NA = not available]
